# Melatonin/Chitosan Biomaterials for Wound Healing and Beyond: A Multifunctional Therapeutic Approach

**DOI:** 10.3390/ijms26135918

**Published:** 2025-06-20

**Authors:** Karolina Kulka-Kamińska, Patrycja Brudzyńska, Mayuko Okura, Tatsuyuki Ishii, Marco Skala, Russel J. Reiter, Andrzej T. Slominski, Kazuo Kishi, Kerstin Steinbrink, Alina Sionkowska, Konrad Kleszczyński

**Affiliations:** 1 Laboratory for Biomaterials and Cosmetics, Nicolaus Copernicus University in Toruń, Gagarin Str. 7, 87-100 Toruń, Poland; kkulka@doktorant.umk.pl (K.K.-K.); patrycja.brudzynska@umk.pl (P.B.); 2 Department of Plastic and Reconstructive Surgery, Keio University School of Medicine, Tokyo 160-8582, Japan; mayuko.okura@keio.jp (M.O.); ttsyksh@keio.jp (T.I.); kkishi@keio.jp (K.K.); 3 Department of Dermatology, University of Münster, Von-Esmarch-Str. 58, 48149 Münster, Germany; uni@skalamarco.eu (M.S.); kerstin.steinbrink@ukmuenster.de (K.S.); 4 Department of Cell Systems and Anatomy, Long School of Medicine, UT Health, San Antonio, TX 78229, USA; reiter@uthscsa.edu; 5 Department of Dermatology, Comprehensive Cancer Center, Cancer Chemoprevention Program, University of Alabama at Birmingham, Birmingham, AL 35294, USA; aslominski@uabmc.edu; 6 VA Medical Center, Birmingham, AL 35294, USA

**Keywords:** chitosan, melatonin, biomaterials, wound healing, anti-cancer therapy, drug delivery systems

## Abstract

Chitosan is increasingly utilized in combination with melatonin in novel formulations for a wide range of therapeutic applications. As a biocompatible and biodegradable polymer, chitosan exhibits notable properties, including antioxidant, antimicrobial, moisturizing, and absorption capabilities, in addition to a high potential for chemical modification due to its functional groups. These characteristics make it a valuable material in biomedical, pharmaceutical, cosmetic, food packaging, and environmental applications. Melatonin, an indoleamine primarily synthesized in the pineal gland but also found in various peripheral organs and in diverse organisms—including plants, bacteria, and fungi—has been extensively investigated for its antioxidant, anti-apoptotic, and anti-inflammatory activities, as well as its roles in immunomodulation, mitochondrial function, and melanin biosynthesis. This review summarizes recent advances in the combined use of chitosan and melatonin, with emphasis on their synergistic effects in wound healing, anti-cancer therapies, tissue engineering (i.e., skin and bone regeneration), and drug delivery systems. Additional potential applications are discussed in the context of cosmetology, aesthetic medicine, and veterinary practice.

## 1. Introduction

**Figure 1:**
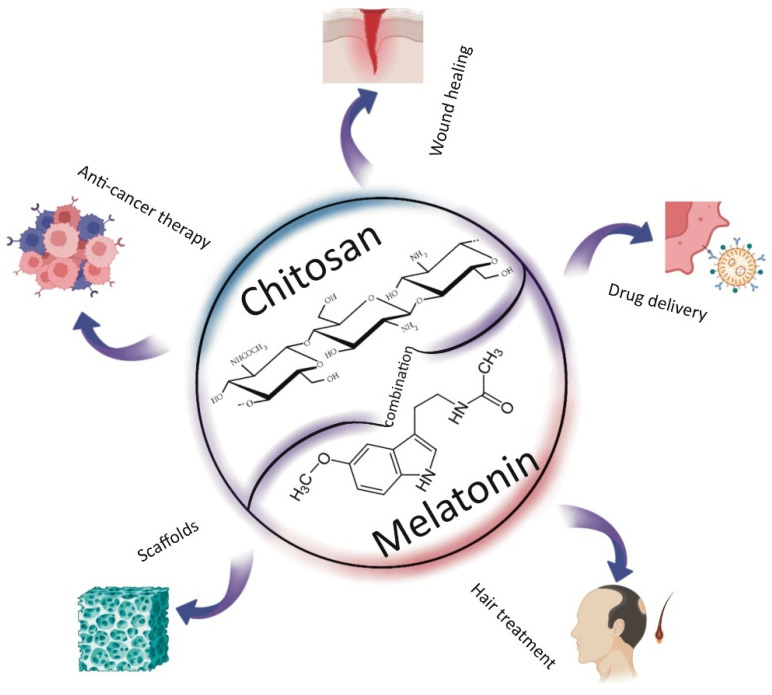
Applications of chitosan and melatonin combinations.

To reduce our environmental impact and dependence on fossil fuels, efforts are increasingly focused on the use of biodegradable materials, particularly those derived from natural sources. Among these, biopolymers stand out for their significant biodegradability and biocompatibility. In recent years, chitosan—a derivative of chitin composed of *β*-(1,4)-*D*-glucosamine and *β*-(1,4)-*N*-acetyl-*D*-glucosamine units—has gained considerable popularity [1,2]. Over the past two decades, chitosan has become a widely used raw material across numerous fields due to its unique properties. Its primary advantages include its proven safety and non-toxicity [3], along with its notable antioxidant and antimicrobial activities. Additionally, chitosan exhibits excellent moisturizing and adsorption capabilities, and its functional groups allow for versatile chemical modifications [4]. However, its application is somewhat limited by its poor solubility in neutral and basic solutions [5]. Chitosan is primarily characterized by two key parameters: molecular weight and degree of deacetylation. The latter should be at least 60% for chitosan to exhibit desirable properties. These parameters significantly influence the physicochemical characteristics of the biopolymer, including its viscosity and solubility—factors that are crucial for its bioactivity, antibacterial and antioxidant properties, biodegradability, toxicity, and biocompatibility [6,7,8]. Low-molecular-weight chitosan typically exhibits better solubility and lower viscosity compared to its high-molecular-weight counterpart. Additionally, it can form denser structures, which may enhance the mechanical properties of materials. According to the literature, low-molecular-weight chitosan also demonstrates improved biodegradability and biocompatibility, lower toxicity, and greater bioactivity than chitosan with a higher molecular weight. Its antioxidant and antimicrobial activities are likewise more pronounced. Therefore, both molecular weight and degree of deacetylation should be carefully considered when designing chitosan-based materials for specific applications [9,10,11]. This biopolymer is widely used across various fields, including medical sciences, pharmacy, cosmetology, and the food and food packaging industries, as well as environmental pollution control [2,12]. It has been approved as a safe substance for use in food and medicine by the United States Food and Drug Administration (US FDA) [4]. Chitosan is particularly valuable as a drug delivery system due to its mucoadhesive properties and enhanced absorption, which stem from its polycationic nature. These characteristics make chitosan an effective matrix for drug encapsulation and delivery [13]. As a carrier, chitosan protects active substances, such as drugs, from oxidation and other environmental factors. For substances sensitive to external conditions, a protective delivery system is essential. Moreover, chitosan-based carriers enable the controlled release of active compounds, a feature that is highly important in many medical treatments. Chitosan is increasingly used in combination with melatonin (*N*-acetyl-5-methoxytryptamine), an indoleamine neurohormone synthesized predominantly in the pineal gland. Melatonin plays a key role in regulating circadian rhythms and a wide range of physiological functions [14]. Its secretion peaks at night but naturally declines with age, a change associated with neurodegenerative processes [15]. Beyond the pineal gland, melatonin is also synthesized in peripheral organs as a protective response against endogenous and exogenous stressors [16,17]. It regulates numerous physiological processes [17] and is found not only in animals but also in plants, bacteria, fungi, and other unicellular organisms [18,19], as well as in several natural products, including honey [20]. Melatonin participates in various cellular processes, exhibiting antioxidant, anti-apoptotic, and anti-inflammatory activities. It also regulates immune responses, mitochondrial homeostasis, and pigmentation [21]. Thanks to its amphiphilic nature, melatonin can act in both lipid and aqueous environments within cells, effectively reducing oxidative stress. It influences glutathione synthesis and the activity of glutathione reductase. Moreover, melatonin has demonstrated potential in anti-cancer strategies [22], and as a promising agent in anti-aging skincare, particularly in mitigating the harmful effects of UV radiation [17,23,24]. However, melatonin has a short half-life and low bioavailability, which limits its therapeutic effectiveness. Encapsulation using biopolymeric carriers such as chitosan offers a promising solution to these limitations [25]. In most applications, chitosan functions as a carrier that protects melatonin until it reaches its target site. Due to its broad biological activity, melatonin is used far beyond sleep regulation, with applications spanning medicine, the food industry, and more. This review compiles and summarizes the scientific findings on the combined use of chitosan and melatonin, focusing on their applications in wound healing, cancer therapy, tissue engineering, drug delivery, and food chemistry, with additional insight into potential uses in cosmetology, veterinary medicine, and agriculture (Figure 1).

## 2. Synergistic Applications of Chitosan and Melatonin

### 2.1. Wound Healing

**Figure 2:**
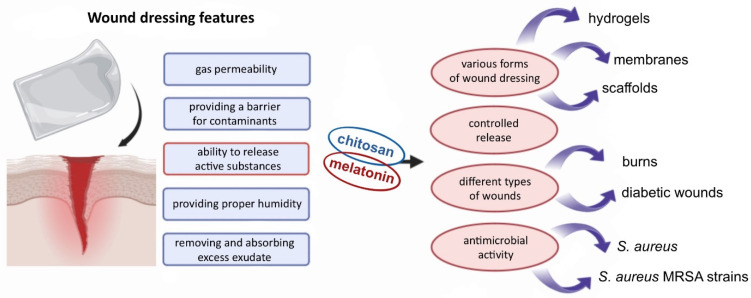
The potential of a chitosan/melatonin combination as a wound-healing agent.

Human skin is constantly exposed to environmental factors and serves as the primary self-regulating barrier against such stressors [17,26]. Beyond its protective function, the skin plays multiple roles, including roles in thermoregulation, vitamin D synthesis, sensory perception, and various secretory functions [27,28,29,30]. As such, any disruption to the skin—such as a wound—can pose a significant threat to human health or, in severe cases, life. Even minor wounds may lead to undesirable outcomes such as scarring. A wound is defined as a disruption of tissue continuity and can be classified based on its cause, type, and treatment duration [27,31]. Wound healing is a complex biological process aimed at restoring the structural and functional integrity of damaged tissue. It occurs through four overlapping phases: hemostasis, inflammation, proliferation, and dermal remodeling [28,32]. This process is tightly regulated and involves dynamic communication between various cell types [33]. Wound dressings play a critical role in supporting and guiding the healing process [25]. The characteristics of an ideal wound dressing include gas permeability, a barrier against external contaminants, absorption and removal of excess exudate, maintenance of optimal moisture levels, and the controlled release of active substances [31,34] (Figure 2). Dressings made from biopolymers are especially promising due to their beneficial properties, including non-toxicity, biodegradability, biocompatibility, and their structural similarity to macromolecules naturally recognized by the human body [31,35].

Modern wound dressings based on biopolymers are designed in various forms, but their primary goal remains the same: to enhance healing efficiency. In this context, nano- and microtechnological solutions have gained considerable interest, particularly those involving microspheres, nanoparticles, and nanofibers. For example, Romic et al. [36] developed melatonin-loaded chitosan-based microspheres as a dry powder formulation suitable for wound dressings. Upon contact with wound exudate, the powder rapidly forms a hydrogel. The encapsulation of melatonin within chitosan/Pluronic F127 microspheres significantly enhanced the antimicrobial activity of chitosan against *Staphylococcus aureus*, including five clinical isolates of methicillin-resistant *S. aureus* (MRSA) strains. These microspheres were also shown to be biocompatible with epidermal keratinocytes and dermal fibroblasts at concentrations relevant to antimicrobial activity against planktonic bacteria. Additionally, the hydrogel formed from these microspheres maintained a favorable water vapor transmission rate (WVTR), ranging from 2357 to 2421 g/m^2^/day, depending on the pH level. This range falls within the optimal WVTR for maintaining wound moisture without dehydration, typically between 2000 and 2500 g/m^2^/day [36]. Chronic, non-healing wounds remain a significant global health challenge. Melatonin has demonstrated potential in promoting diabetic wound healing and supporting corneal regeneration. In a subsequent study, Romic et al. [37] evaluated two types of melatonin-loaded chitosan microspheres. The research included full characterization of the microspheres, stability testing, and assessment of their wound-healing potential. Results showed that the chitosan microspheres effectively protected melatonin from degradation, even after six months of storage. The wound-healing properties of the formulations were evaluated using an in vitro scratch assay with human dermal fibroblasts. While both microsphere systems exhibited good stability, the inclusion of lipids enhanced their wound-healing efficacy [37]. Romic et al. [38] developed a hybrid wound-dressing system composed of nanostructured lipid carrier (NLC)-loaded, chitosan-based microspheres containing melatonin in dry powder form. This advanced formulation is designed to form a hydrogel layer upon contact with wound exudate, providing a modern approach to wound care. Nanostructured lipid carriers serve as an effective matrix for active substances, enabling controlled drug release. The preparation process involved two key steps: first, melatonin-loaded NLCs were produced using a hot homogenization technique; second, the final dry powder was obtained through spray drying. The release profile of melatonin from this hybrid system followed a biphasic pattern characterized by an initial burst release followed by a sustained-release phase. This dual-phase release is beneficial for wound healing, as it allows for immediate antimicrobial action while maintaining therapeutic levels of the drug over an extended period. The formulation demonstrated biocompatibility with human keratinocytes and fibroblasts, indicating its safety for topical use. Moreover, the system exhibited antimicrobial activity against *Staphylococcus aureus* and MRSA strains [38,39]. Cytotoxicity studies further confirmed the safety of the nanoparticle-based system, showing no membrane damage or reduction in cell viability even at concentrations up to 200 μg/mL.

Melatonin is considered a strong candidate for transdermal delivery due to its low molecular weight, amphiphilic chemical nature, and short plasma half-life. Topical application of melatonin is also desirable because of its anti-inflammatory and antioxidant properties [39]. In a study by Blazevic et al. [40], an alternative strategy was developed involving the use of nanocarriers to locally deliver melatonin for wound-healing purposes. Lecithin/chitosan nanoparticles were prepared using four different types of chitosan, all in the form of hydrochloride salts with varying molecular weights and degrees of deacetylation. Two of the types of chitosan had molecular weights ranging from 50 to 150 kDa, while the other two ranged from 150 to 400 kDa. The degree of deacetylation was either between 75 and 90% or greater than 90%. An in vitro scratch assay was used to evaluate the wound epithelialization potential. Nanoparticles derived from chitosan with a molecular weight of 50–150 kDa and a deacetylation degree above 90% demonstrated the highest wound-healing efficacy. However, the differences compared to other samples were not statistically significant. Therefore, it can be concluded that the various types of chitosan used in nanoparticle formulation have comparable effects on keratinocyte proliferation and migration. The key factor enhancing wound epithelialization appears to be the combination of chitosan and melatonin within the nanoparticle suspension, which may exert a synergistic effect [40]. Kaczmarek-Szczepańska et al. [41] developed biomimetic hybrid scaffolds based on a biopolymer matrix containing not only chitosan but also collagen—a valuable material for tissue engineering—enriched with melatonin for potential wound-dressing applications. The prepared scaffolds significantly enhanced cell viability in reference melanoma cells, dermal fibroblasts, and epidermal keratinocytes. Materials containing melatonin demonstrated accelerated re-epithelialization, increased water retention, and reduced wound-healing duration. Furthermore, the presence of this biologically active compound did not adversely affect the thermal stability of the scaffolds [41]. As previously mentioned, wound care in diabetic patients is a critical global health concern, often leading to amputations and even death. Correa et al. [42] developed an in vivo animal model to evaluate the wound-healing potential of lecithin/chitosan nanoparticles loaded with melatonin in the context of diabetes. The nanoparticles exhibited a positive surface charge due to the presence of chitosan—an important characteristic for wound dressings, as it facilitates interaction with cells in the wound environment. In a rat wound-healing model, treatment with melatonin-loaded nanoparticles for three days significantly stimulated fibroblast proliferation and increased the number of blood vessels, indicating that this formulation promotes healing at the early proliferative phase. Similar results were observed after seven days of treatment. Additionally, an increase in collagen content was noted in the rats treated with the melatonin-loaded chitosan nanoparticles. This formulation effectively protected encapsulated melatonin and ensured its controlled delivery to the wound site. The nanoparticle structure enabled sustained release of the active biomolecule, contributing to the angiogenesis process [42]. 

Burn wounds are a distinct type of skin injury that require targeted treatment to prevent infections and minimize scarring. Traditional burn treatments often rely on silver-based formulations due to their antimicrobial properties; however, the cytotoxicity of silver compounds can delay the healing process. Melatonin, known for its antioxidant and anti-inflammatory properties, presents a promising alternative for this application. In a study conducted by Soriano et al. [43], a nanogel formulation based on hyaluronic acid, chitosan, and poloxamer, with the addition of melatonin, was developed. This formulation was compared with a commercially available silver-based preparation. Cytotoxicity assays revealed that the melatonin-containing nanogel was non-toxic and exhibited excellent physicochemical and wound-healing properties while also being biocompatible with healthy skin [43]. A different approach was proposed by Mirmajidi et al. [44], who developed a more complex multilayered wound dressing using the electrospinning technique. The dressing consisted of three layers: the outer and inner layers were composed of polycaprolactone/chitosan, and the middle layer was made of poly(vinyl alcohol) containing melatonin. The resulting nanofiber dressing was found to be nearly hydrophilic, a characteristic not affected by melatonin concentration. This hydrophilicity is advantageous for maintaining a moist wound environment, which supports the healing process. The multilayer dressing functioned as both a reservoir and a matrix for drug release. It exhibited an initial burst release of melatonin during the first 14 h, followed by a sustained release over time—an ideal profile for wound patches intended to remain in place for several days. In vivo studies confirmed the dressing’s positive impact on wound healing, showing enhanced collagen synthesis and improved regeneration of the epithelial layer [44].

In addition to their proven efficacy in wound healing, systems combining chitosan and melatonin offer another significant advantage: the protection of melatonin during storage prior to application. Hafner et al. [45] investigated the stability of lecithin/chitosan nanoparticles loaded with melatonin following a freeze-drying process. The study evaluated the effects of different types of lecithin and various cryoprotectants, including glucose and trehalose. The results demonstrated that trehalose was the more effective cryoprotectant, enabling the storage of melatonin-loaded chitosan-based nanoparticles for up to seven months at 4 °C without significant changes in appearance, physicochemical properties, or melatonin content [45]. Melatonin release behavior is a critical factor in many biomedical applications. Pancescu et al. [46] conducted a detailed analysis of melatonin release through chitosan/sEPDM (sulfonated ethylene/propylene/diene terpolymer) composite membranes under various conditions. Compared to pure sEPDM membranes, the composite membranes exhibited a faster melatonin release rate. The authors suggested that this system may be particularly suitable for sports-related applications, where rapid drug action is essential [46]. Beyond wound dressings, the combination of chitosan and melatonin is showing increasing promise in biomedical engineering, particularly in the development of scaffolds for tissue regeneration and repair.

### 2.2. Scaffolds and Hydrogels

According to the World Health Organization, only 10% of the global demand for tissues and organs is currently met, representing a significant public health challenge [47]. Advances in regenerative medicine and tissue engineering offer promising solutions to address this shortage. The primary goals of tissue engineering include restoring, replacing, maintaining, or enhancing the function of various types of biological tissues [48]. With the rapid progress in this field, scaffolds have gained considerable attention, particularly those made from natural polymers. Chitosan is one such biopolymer widely used in scaffold construction for applications such as bone regeneration [48,49,50], oral bone repair [51], cardiovascular treatments [52,53], and skin regeneration [54,55]. Recently, chitosan-based scaffolds combined with melatonin have emerged as a novel and promising approach in tissue engineering. Bone regeneration relies on multiple factors, with bone morphogenetic proteins (BMPs) playing a crucial role. Among them, BMP-2 is the most well-known and potent osteoinductive agent. However, the use of high doses of BMP-2 in bone treatment can lead to undesirable side effects, such as osteoclast activation.

In a study conducted by Jarrar et al. [56], scaffolds composed of chitosan and hydroxyapatite were developed with the addition of BMP-2 and melatonin, with the latter being used as an osteoclast-inhibiting agent at a concentration of 800 μM. This concentration of melatonin effectively attenuated osteoclast differentiation induced by a high dose of BMP-2, suggesting that the designed scaffolds hold significant promise for bone regeneration therapies [56]. In a subsequent study by the same research group [57], the focus was on evaluating the osteogenic activity of pre-osteoblastic MC3T3-E1 cells in response to the prepared scaffolds. The tested material consisted of a chitosan/hydroxyapatite scaffold loaded with polylactic-co-glycolic acid (PLGA) microparticles encapsulating both melatonin and BMP-2. These microparticles were synthesized using a double emulsion solvent evaporation method, resulting in a dual-release scaffold system. The study demonstrated that the synergistic effect of the combined release of melatonin and BMP-2 significantly enhanced the osteogenic activity of pre-osteoblasts in vitro, compared to single-agent systems containing either melatonin or BMP-2 alone. The dual system notably increased the expression of early osteogenic markers and promoted osteoblast formation [57,58].

As previously mentioned, melatonin has demonstrated anti-cancer properties, including anti-proliferative effects and the ability to inhibit tumor promotion and progression. One study investigated the formation of melatonin inclusion complexes by incorporating melatonin into the hydrophobic cavity of cyclodextrins, a strategy aimed at enhancing melatonin’s solubility. Cell culture experiments were conducted using three types of chitosan-based scaffolds: a plain chitosan scaffold, a scaffold loaded with melatonin, and a scaffold loaded with the melatonin/cyclodextrin inclusion complex. The cumulative release of melatonin from the inclusion complex was higher than from the scaffold containing pure melatonin. In vitro studies showed that approximately 5.1 mM of melatonin was released from the melatonin-loaded scaffold, whereas 9.3 mM was released from the inclusion complex scaffold. A melatonin concentration of 9 mM was sufficient to induce significant cell growth inhibition, while a 5 mM concentration initially caused some cell death, but surviving cells resumed proliferation over the remainder of the study period [58]. Another research group proposed a scaffold-based device incorporating melatonin for osteosarcoma therapy, evaluating its dual activities. First, a rapid release of melatonin at high concentration was intended to exert anti-cancer effects, then a sustained low-concentration release was intended to promote bone regeneration. To investigate the first approach, the effect of a melatonin/2-hydroxypropyl-β-cyclodextrin (HPβCD) inclusion complex on MG-63 human osteosarcoma cells was assessed. Melatonin was rapidly released within 5 h, leading to inhibited cell proliferation by arresting cells in the G0/G1 phase. The second approach involved incorporating melatonin into poly(lactic-co-glycolic acid) (PLGA) microparticles. Both delivery systems were then loaded into chitosan/hydroxyapatite scaffolds, enabling controlled melatonin release characterized by an initial burst within 24 h followed by sustained release over 40 days at concentrations ranging approximately from 40 to 70 μM. In this sustained-release scenario, melatonin significantly increased the expression of osteogenic differentiation markers compared to controls. This scaffold system thus exhibits both anti-cancer and osteoinductive properties, making it a promising therapeutic option for osteosarcoma patients, particularly post-surgery, to eliminate residual cancer cells and promote new bone formation [59]. In a related study, Huang et al. [60] aimed to prepare melatonin-loaded chitosan microparticles with osteoinductive and osteoconductive potential. Two fabrication methods were employed: ionic cross-linking and an oil-in-water emulsion technique. The in vitro release profiles of melatonin were comparable between the two types of microparticles, and mineralization matrix formation assays were conducted. Results demonstrated that this sustained-release system effectively induced osteogenic differentiation in vitro. Alkaline phosphatase, an early osteoblast differentiation marker, was activated through continuous melatonin administration, which also promoted calcium deposition. This controlled-release platform may thus offer beneficial effects for bone tissue regeneration [60].

In the study by Kaczmarek-Szczepańska et al. [61], 3D skin scaffolds composed of chitosan and collagen and loaded with melatonin were developed. Glyoxal was used as a cross-linking agent to achieve optimal porosity, as well as favorable physicochemical and mechanical properties. Various biopolymer ratios were prepared, both with and without glyoxal. The results demonstrated that melatonin enhanced cell proliferation regardless of whether the cross-linker was present. Increased growth rates were observed in human keratinocytes, fibroblasts, and melanoma cells. Melatonin contributed to maintaining mitochondrial homeostasis, protecting cells against reactive oxygen species, and supporting the skin’s barrier function by stimulating the expression of involucrin and keratins. The porous structure of the scaffolds was a critical feature, allowing oxygen flow and thereby accelerating tissue repair. Overall, the melatonin-loaded chitosan/collagen scaffolds showed promise as safe and effective materials for skin restoration applications [61].

### 2.3. Cosmetology/Dermatology (Hair Treatment)

Melatonin has also been explored for its potential applications in cosmetics and dermatology, particularly in the treatment of androgenic alopecia. This genetically predisposed, chronic condition is characterized by a shortened anagen phase and a prolonged telogen phase within the hair growth cycle. Hair loss associated with androgenic alopecia can significantly impact quality of life and self-esteem. Currently, the U.S. Food and Drug Administration (FDA) has approved two treatments: orally administered finasteride and topically applied minoxidil [62]. However, both agents are associated with adverse effects and often demonstrate limited efficacy, prompting the need for alternative therapies. Melatonin emerges as a promising candidate in this context. Human skin and hair follicles contain melatonin receptors, which play a role in regulating cellular proliferation and differentiation [16,62]. Moreover, melatonin and its metabolites exert bioregulatory effects through nuclear receptors such as the aryl hydrocarbon receptor (AhR) and peroxisome proliferator-activated receptor gamma (PPARγ) [63]. The skin also possesses the enzymatic machinery required to synthesize melatonin from tryptophan via a series of enzymatic reactions [16]. Melatonin contributes not only to hair growth regulation but also to hair pigmentation [64]. Additionally, the skin’s endogenous melatoninergic antioxidant system offers protection against UV-induced damage [16,65]. A study by Fischer et al. [64] investigated the topical application of melatonin in patients with androgenic alopecia (AGA) or general hair loss. Results demonstrated a significant reduction in the severity of alopecia after 30 and 90 days of once-daily application. Importantly, topical melatonin did not affect endogenous serum melatonin levels. The authors concluded that the melatonin solution presents a viable treatment option for AGA [62]. Melatonin’s efficacy in treating alopecia is attributed to its antioxidant properties and biochemical activity at the levels of corneocytes and hair follicles. However, melatonin is susceptible to photodegradation and oxidation, which can limit its effectiveness. To address this, Elshall et al. [66] encapsulated melatonin in Pickering emulsions stabilized with chitosan/dextran sulfate nanoparticles. The formulation was compared with minoxidil, the standard drug used for alopecia treatment. These chitosan/dextran sulfate-based emulsions enhanced the photostability of melatonin and preserved its antioxidant activity [66].

Overall, formulations combining chitosan and melatonin offer a promising alternative for the treatment of androgenic alopecia, with potential advantages in stability, biocompatibility, and therapeutic efficacy.

### 2.4. Anti-Cancer Therapy

**Figure 3:**
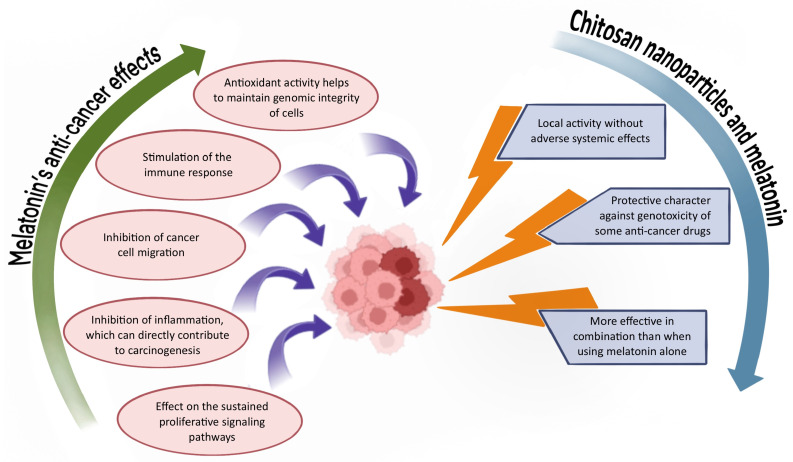
Overview of the properties and benefits of the chitosan/melatonin combination in anti-cancer therapy.

Cancer remains one of the most prevalent diseases worldwide, resulting from uncontrolled cellular proliferation [67,68]. A variety of treatment modalities are currently employed, including chemotherapy, radiation therapy, immunotherapy, targeted therapies, and surgical resection [67,68]. While conventional treatments such as chemotherapy can be effective, cancer-related mortality rates remain high. Major limitations of these therapies include a lack of specificity, poor drug solubility, and significant cytotoxicity to healthy tissues [68]. Emerging anti-cancer strategies rooted in precision medicine are increasingly based on nanotechnology [69]. Nanoparticles offer several advantages over traditional therapies, including localized activity that reduces systemic side effects. In addition, they are typically non-toxic, biodegradable, biocompatible, and non-immunogenic, and they can facilitate the delivery of poorly soluble drugs [69]. Researchers are also actively exploring natural substances as potential anti-cancer agents [70]. Among these, melatonin has gained significant interest due to its multifaceted biological activity [22]. Although further studies are needed to fully elucidate its mechanisms of action in cancer treatment [71], melatonin is recognized for its anti-cancer potential through various pathways. These include modulation of cell signaling pathways, antioxidant effects, protection of genomic integrity, inhibition of cancer cell migration, and suppression of the inflammatory processes associated with carcinogenesis [72,73,74]. The combination of chitosan nanoparticles and melatonin is gaining increasing attention in cancer research, as illustrated in Figure 3.

Breast cancer is the second most common type of cancer among women worldwide. Although various treatment options are available, resistance to therapy remains a significant clinical challenge. A novel approach was proposed by Jafari et al. [75], who developed composite nanoparticles incorporating melatonin for potential use in breast cancer therapy. The polymer matrix consisted of chitosan and hydroxypropyl methylcellulose, cross-linked with tripolyphosphate. The release profile of melatonin from the matrix varied depending on the pH of the environment, with significantly higher cumulative release observed at pH 5.5 compared to pH 7.5. Both free and encapsulated melatonin demonstrated cytotoxicity toward MDA-MB-231 breast cancer cells; however, encapsulated melatonin showed enhanced toxicity. These findings suggest that the proposed composite nanoparticle system may be more effective in acidic tumor microenvironments [75]. Another research group, led by Yadav et al. [76], developed chitosan nanoparticles loaded with melatonin as a potential anti-cancer therapy. The nanoparticles were prepared using the ionic gelation method with a tripolyphosphate solution. Cytotoxicity studies were conducted using the U87MG cell line, a human glioblastoma model. After 24 h of incubation, melatonin-loaded nanoparticles reduced cell viability to 22%, a significantly better outcome compared to the control. To assess safety for healthy cells, the HEK293T cell line was used, and the results indicated very low cytotoxicity. Furthermore, co-culture experiments with both HEK293T and U87MG cells demonstrated moderate overall cytotoxicity. These findings highlight the potential of this nanoparticle system for glioblastoma treatment [76].

Melatonin is recognized not only for its anti-cancer properties but also for its role as an organ protectant during cancer pharmacotherapy [22]. Shokrzadeh et al. [77] conducted studies demonstrating melatonin’s protective effect against genotoxicity induced by etoposide—a potent chemotherapeutic agent known for its side effects, including vomiting, alopecia, and DNA damage in healthy tissues. In this study, the protective effects of both free melatonin and melatonin in nanoparticle form were evaluated using human HepG2 hepatoma cells. Melatonin was encapsulated in nanoparticles using chitosan and sodium tripolyphosphate. The results showed that both forms of melatonin conferred protection against etoposide-induced genotoxicity, but the nanoparticle formulation was more effective. A concentration of 100 μmol/L significantly reduced DNA fragmentation in the treated cells [77]. In another study by the same research group, Shokrzadeh et al. [25] investigated the protective properties of chitosan/tripolyphosphate nanoparticles loaded with melatonin against the genotoxic effects of doxorubicin, another widely used chemotherapeutic drug. Melatonin was utilized for its known genoprotective and antioxidant effects. The results revealed that melatonin significantly reduced doxorubicin-induced genotoxicity, lowered intracellular reactive oxygen species levels, and increased glutathione content in HepG2 cells. These findings suggest that melatonin-loaded nanoparticles, prepared via the ionotropic gelation method, offer substantial protective effects against chemotherapy-induced cellular damage [25].

Overall, numerous studies underscore the broad potential of chitosan-based systems enriched with melatonin in addressing cancer-related challenges, both as therapeutic agents and as supportive protective strategies.

### 2.5. Drug Delivery Systems

Chitosan is a chemically stable compound that is biocompatible with a wide range of tissues and cells [78]. Owing to its beneficial properties—including its antimicrobial, antioxidant, anti-inflammatory, and mucoadhesive effects, as well as its ability to facilitate controlled drug release, in situ gelation, and enhanced permeation—it is considered a highly suitable biopolymer for use in drug delivery systems. These favorable characteristics are primarily attributed to the presence of primary amino groups, which confer chitosan’s cationic nature [79]. Chitosan can be utilized via various delivery routes, including nasal, oral, transdermal, pulmonary, ocular, vaginal, and ophthalmic routes, depending on its formulation [78,80]. Among these, nanoparticles are the most commonly used form in drug delivery applications. Chitosan nanoparticles are advantageous due to their effectiveness, ease of preparation, and enhanced stability [80]. Methods for producing chitosan nanoparticles include ionic cross-linking, covalent cross-linking, the reverse micellar method, precipitation, and the emulsion-droplet coalescence method [80,81]. Importantly, chitosan nanoparticles are generally considered safe and exhibit low toxicity. However, some studies have reported that the toxicity of unmodified chitosan may increase with higher charge density and a greater degree of deacetylation [82,83]. Overall, chitosan serves as a versatile drug carrier which is particularly suitable for transporting sensitive pharmaceutical substances, such as melatonin, that require protection from external factors.

In the study conducted by Hafner et al. [84], two types of nanocarriers were developed for ocular drug delivery of melatonin. The first formulation consisted of lecithin/chitosan nanoparticles, while the second involved Pluronic 127/chitosan micelles. The nanoparticles were larger in size and exhibited a higher surface charge compared to the micelles. The mucoadhesive properties of the lecithin/chitosan nanoparticles were also evaluated, as these characteristics are critical for prolonging drug residence time on the ocular surface. Chitosan-based nanoparticles demonstrated enhanced mucoadhesive capabilities, which correlated with their higher surface charge. Cytotoxicity was assessed using HCE-T cells treated with chitosan concentrations ranging from 2.5 to 20 μg/mL. Cell viability assays indicated that the chitosan-based nanosystems were safe, showing no significant toxic effects. Notably, chitosan incorporated into nanocarriers was less cytotoxic than chitosan in solution form, highlighting the advantage of nanoparticle formulation. In vitro tests using a corneal epithelial model showed that lecithin/chitosan nanoparticles provided a prolonged release of melatonin compared to both the micelle system and melatonin solution. These properties are promising for ocular applications, offering improved bioavailability and potential for intraocular pressure reduction [84].

Novel hydrogel materials based on chitosan, incorporating melatonin or propolis as antioxidant agents along with other active substances, were evaluated for use in stomatology by Perchyok et al. [85]. The antioxidants, including melatonin, were microencapsulated. The stability of these antioxidants within the system was assessed after 24 h and again after 6 months of storage using a cupric ion (Cu^2+^) reducing strength assay. The results showed over 95% stability, indicating effective protection by the microcarriers. Additionally, the presence of chitosan enhanced the antioxidant activity, suggesting a synergistic effect between the biopolymer and the antioxidants. The proposed hydrogel material also improved dentin adhesive bond strength and positively influenced drug release behavior [85].

Excessive reactive oxygen species (ROS) can impair oocyte maturation, often due to insufficient antioxidant defenses. In a study by Tawfik et al. [86], melatonin supplementation was used in maturation media to investigate its antioxidant effects on the cytoplasmic and nuclear maturation of porcine oocytes. Melatonin also acted as a cytoprotective agent, preventing nitrosative damage. However, its clinical application is challenged by its short half-life and low water solubility. Nanocapsules offer a promising solution for effective melatonin delivery. The study revealed significant differences in gene expression profiles among the groups treated with melatonin-loaded chitosan nanoparticles, free melatonin, and the control. Encapsulated melatonin nanoparticles proved to be the most effective in enhancing oocyte maturation [86].

Chitosan nanoparticles have been used as carriers for melatonin due to its anti-inflammatory properties. In a study by Soni et al. [87], melatonin-loaded chitosan nanoparticles were evaluated for their potential in treating inflammatory bowel disease (IBD). The primary goal was to enhance the drug release profile and assess the therapeutic efficacy of the formulation. The synthesized nanoparticles demonstrated improved anti-inflammatory activity in both in vitro and in vivo IBD models, confirming their protective and therapeutic effects. Consequently, the therapeutic efficacy of melatonin was enhanced [87]. In a separate study, Mohanbhai et al. [88] also investigated the use of melatonin and chitosan for IBD treatment. Given melatonin’s poor water solubility, the researchers developed chitosan nanoparticles coated with a colon-targeting polymer (Eudragit S-100) to enhance its delivery. The formulation enabled prolonged and targeted release of melatonin in the colon, resulting in improved therapeutic effectiveness [88]. Additionally, chitosan was employed as a nanocarrier for the co-delivery of melatonin and tretinoin using a self-assembly method. Aghaz et al. [89] evaluated the antioxidant activity of this dual-drug system in a mouse oocyte and embryo model. The results indicated that the combination effectively reduced ROS levels and exhibited synergistic antioxidant effects. Taken together, the existing literature demonstrates that chitosan is an effective carrier for melatonin, enhancing its stability, bioavailability, and therapeutic potential across various biomedical applications.

### 2.6. Other Applications

**Figure 4:**
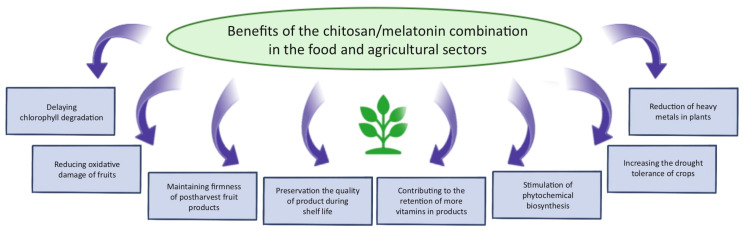
Summary of the benefits of using a chitosan/melatonin combination in the food industry and agriculture.

Melatonin has a wide range of applications beyond medicine and dermatology, including uses in jet lag treatment, food technology, and agriculture. Jet lag is a condition characterized by disturbances in the sleep–wake cycle which is often accompanied by fatigue, impaired cognitive performance, daytime sleepiness, and gastrointestinal discomfort. These symptoms result from rapid transitions across time zones and can persist for several days, significantly affecting the quality of life of travelers and long-haul flight crews [90]. Melatonin supplementation, taken at times when it would naturally be released by the body, can facilitate adaptation to new time zones and alleviate jet lag symptoms [91]. In an effort to address this issue, Razali et al. [92] developed a multi-particulate delivery system incorporating both melatonin and caffeine. The objective of the study was to design a dual-release formulation: one that sustains the release of melatonin to promote sleep and another that delays the release of caffeine to support wakefulness after the sleep period. Various system configurations were evaluated, including pellets, compact forms, and a dome-shaped matrix. The most effective melatonin delivery system consisted of an alginate/chitosan matrix with a cushioning agent—low-viscosity hydroxypropyl methylcellulose—which enabled complete gastrointestinal release of melatonin within 8 h. For caffeine, the most promising delayed-release system included microcrystalline cellulose, high-viscosity hydroxypropylcellulose, and high-viscosity ethylcellulose. This combination successfully delayed caffeine release until approximately 8 h after melatonin administration, aligning with the desired timing for post-sleep alertness [92]. The applications of chitosan in food technology stem from its advantageous physicochemical properties, biological activity, and safety profile. Key areas of its utilization include shelf-life extension, functional food production, emulsification, and flocculation [1] (Figure 4). The extension of food shelf life is primarily attributed to chitosan’s antioxidant, antimicrobial, and film-forming properties. Edible films and coatings made from chitosan represent a simple and effective packaging method, which can be further enhanced with the addition of flavorings, colorants, preservatives, and other active ingredients [1,93]. The combination of chitosan and melatonin in food-related applications offers synergistic effects, particularly in enhancing antioxidant potential. Melatonin plays a significant role in preserving the quality of food products by delaying chlorophyll degradation, reducing oxidative damage in freshly cut fruits, minimizing weight loss, and maintaining firmness in postharvest fruit products [94]. This combination presents a promising strategy for improving the quality and extending the shelf life of fresh produce.

Al-Quarashi et al. [95] proposed a postharvest dipping treatment for bananas that used 1% chitosan and 0.5 mM melatonin, applied both individually and in combination. The treatment delayed ripening by preserving a greener peel and greater firmness compared to the control group. Additionally, treated bananas exhibited higher total phenolic and flavonoid content, as well as enhanced antioxidant activity [95]. The same research group later evaluated the effects of a similar postharvest dipping treatment for mature green limes over a 20-day storage period. As limes undergo various metabolic changes during storage—such as color fading—the study investigated the effects of melatonin and chitosan both separately and together. Results demonstrated that these treatments enhanced the fruit’s antioxidant system and helped retain quality for up to 16 days of shelf life. The melatonin treatment preserved more vitamin C compared to the control; however, no synergistic benefits were observed when melatonin and chitosan were used in combination [96]. Similarly, Bal et al. [97] studied the effects of chitosan coatings enriched with melatonin on the storability of sweet cherries. Weekly assessments included assessment of parameters such as weight loss, firmness, and anthocyanin, phenolic, and antioxidant content. The results showed that the chitosan/melatonin composite coating reduced respiration rate and weight loss while preserving higher biochemical content and fruit firmness [97]. Zhao et al. [94] evaluated a chitosan-based melatonin coating applied to fresh-cut produce, including cucumber (*Cucumis sativus*), broccoli (*Brassica oleracea*), and melon (*Cucumis melo* var. *saccharinus*). The coating delayed chlorophyll degradation and improved antimicrobial and antioxidant properties [94].

In another study, chitosan and melatonin were used to create a composite coating for pomegranate aril sacs to delay aging and maintain quality. The sacs were immersed in the coating solution, air-dried at room temperature, and stored at 5 °C for 21 days. The treatment reduced weight loss and respiration rate, and resulted in higher anthocyanin content, antioxidant activity, and chroma values. Moreover, it helped to minimize surface browning, indicating its effectiveness in preserving pomegranate aril quality [98]. Ullah et al. [99] investigated the use of chitosan and melatonin as elicitors in agitated shoot cultures of *Ajuga integrifolia*. Elicitors are agents that induce physiological stress to enhance phytochemical biosynthesis. Antioxidant activity was assessed using DPPH, ABTS, and FRAP assays. Melatonin significantly enhanced antioxidant activity and stimulated the biosynthesis of several phytochemicals, including chlorogenic acid, rosmarinic acid, apigenin, and quercetin. Chitosan also showed notable effects, particularly on anti-inflammatory activity [99]. In agriculture, melatonin is recognized for its role in enhancing plant drought tolerance. To protect melatonin from environmental degradation, Zhou et al. [100] encapsulated it in hybrid particles composed of chitosan, sodium tripolyphosphate, and pectin. These particles, designed to release melatonin gradually, were tested on wheat seedlings. SEM imaging revealed that the particle surfaces were rougher than pure melatonin, and photodegradation was significantly reduced. While chitosan did not affect seedling growth, the encapsulated melatonin increased dry weight, root length, and peroxidase activity, demonstrating improved drought resistance [100]. Cadmium accumulation in crops poses a global agricultural challenge. Chen et al. [101] developed a foliar spray delivery system using mesoporous silica nanoparticles and chitosan/mesoporous silica nanoparticles to administer melatonin. Both carriers enabled controlled release, but the chitosan-modified system performed better in vitro. In rice plants, this nano-delivery system significantly reduced cadmium accumulation, increased antioxidant enzyme activity, and enhanced photosynthetic efficiency, outperforming free melatonin and melatonin with silica alone [101]. Additionally, Tabassum et al. [102] studied the effects of chitosan foliar treatment on two accessions of *Pisum sativum* L. The biopolymer treatment improved antioxidant activity, plant growth, and inorganic ion accumulation, and also stimulated endogenous melatonin synthesis in the tested plants [102].

Chitosan and melatonin have also found promising applications in veterinary medicine. López-Valverde et al. [103] evaluated bone density around dental implants in canine jaws, comparing standard etched implant surfaces with those coated with chitosan and melatonin. After 12 weeks, no statistically significant differences were found in bone density between the groups. This lack of difference may be attributed to the mechanical instability of the surface coatings, which might have been lost during implantation due to low mechanical resistance [103]. A more successful application was demonstrated by Abdelrasoul et al. [104], who developed a composite hydrogel composed of alginate, chitosan, and β-tricalcium phosphate loaded with melatonin. The hydrogel’s regenerative capacity was tested in six dogs with periodontal defects. After eight weeks, the melatonin-loaded scaffold enhanced bone formation and supported complete periodontal regeneration. The newly formed bone showed quality comparable to native compact bone [104]. In a subsequent study, Abdelrasoul et al. [105] further assessed the hydrogel’s systemic toxicity, physical effects on bone defects, and regenerative performance using a rabbit model. Both the melatonin-loaded and unloaded scaffolds were found to be biocompatible, with no signs of systemic toxicity. While both groups showed reductions in defect depth, volume, and area size, the overall rate of regeneration was similar between scaffolds with and without melatonin over an 8-week period [105]. Beyond biological and clinical applications, chitosan has shown potential as a functional material in melatonin detection systems. Chitosan, in combination with nano-carbon acetylene black, has been used to construct a sensor capable of the simultaneous detection of melatonin and serotonin in biological samples [106]. In another study, a screen-printed carbon electrode modified with nano-ceria and chitosan enhanced electrochemical sensitivity, providing a selective, fast, and sensitive method for melatonin determination [107]. Similarly, a composite sensor based on a glassy carbon electrode modified with chitosan and carboxylated multi-walled carbon nanotubes enabled simultaneous detection of melatonin, serotonin, and dopamine in human saliva samples [108].

These studies highlight the versatility of chitosan/melatonin-based materials, which not only serve in wound healing, bone regeneration, and disease treatment in both human and veterinary medicine but also show promise as components in analytical tools for hormone detection. The broad spectrum of applications is summarized in Table 1.

## 3. Conclusions and Perspectives

**Figure 5:**
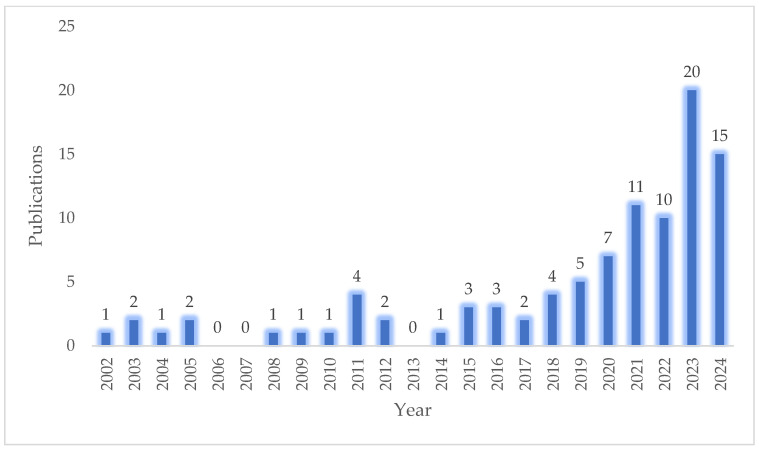
Publications on the subjects of melatonin and chitosan over the years (search for words “melatonin” and “chitosan” in the title, abstract, and keywords). The figure is based on data from the Scopus database [accessed on 12 December 2024].

The health and life sciences sector is constantly evolving, driven by emerging challenges and the need for innovative therapeutic strategies. A common approach involves combining well-established bioactive compounds to enhance efficacy and broaden their applicability. A review of the Scopus database reveals that research on the chitosan/melatonin combination has been ongoing since the early 2020s. A keyword search for ‘melatonin’ and ‘chitosan’ yields 96 research articles published between 2002 and 2024 (Figure 5). Although this is not yet a widely explored topic, the growing number of publications indicates a rising interests in this synergistic pairing, likely due to its diverse range of potential applications. Melatonin, best known for its role in circadian rhythm regulation via pineal gland secretion, is also synthesized in peripheral organs where it exerts various local regulatory functions. Its therapeutic versatility—including antioxidant, anti-inflammatory, immunomodulatory, and anti-cancer activities—makes it a valuable candidate for treating neurodegenerative disorders, immune-related conditions, and malignancies. Despite its biocompatibility and safety, melatonin’s instability and rapid degradation in physiological environments necessitate the use of protective delivery systems, particularly at the nanoscale [109]. Chitosan, a natural polysaccharide derived from chitin, offers multiple advantages as a carrier for melatonin, including mucoadhesive properties, enzymatic resistance, and inherent antimicrobial and antioxidant activity. The functional versatility of chitosan enables the development of innovative delivery platforms that enhance melatonin’s stability and bioavailability. The scientific evidence reviewed in this article demonstrates that chitosan can effectively serve as a carrier or enhancer for melatonin, enabling both systemic and topical therapeutic applications. The integration of melatonin with chitosan has opened up new possibilities in tissue engineering, wound healing, cancer treatment, veterinary medicine, food preservation, and cosmetics (Table 1). Although research on chitosan/melatonin formulations is still in its early stages, preliminary findings are very promising. This combination represents a compelling frontier in biotechnology, with significant potential for expansion. The recent increase in related publications suggests a growing recognition of this pairing’s utility. Future advancements are likely to focus on surface-functionalized biomaterials and nanotechnology-based delivery platforms. As the field matures, novel and currently unforeseen applications are expected to emerge, further solidifying the role of chitosan/melatonin systems in advanced therapeutic and industrial innovations.

## Figures and Tables

**Table 1:** Chitosan/melatonin-based materials and their applications.

Chitosan/Melatonin Combination				
Application	Matrix Composition	Form/System	Year	Reference
Wound healing	chitosan/Pluronic^®^ F127	microspheres	2016	[36]
	chitosan/Pluronic^®^ F127	microspheres enriched with nanostructured lipid carriers	2019	[37,38]
	lecithin/chitosan	nanoparticles	2011	[39]
	lecithin/chitosan	nanoparticles	2016	[40]
	chitosan/collagen	scaffold	2021	[41]
	lecithin/chitosan	nanoparticles	2020	[42]
	Poloxamer407/chitosan/ hyaluronic acid	nanogel	2020	[43]
	chitosan–polycaprolactone/ polyvinyl alcohol–melatonin/ chitosan–polycaprolactone	three-layer nanofiber	2021	[44]
	lecithin/chitosan	nanoparticles	2011	[45]
	chitosan/sulfonated ethylene-propylene-diene terpolymer (sEPDM)	membrane	2023	[46]
Scaffolds and hydrogels	chitosan/hydroxyapatite (HAp)	scaffold	2021	[57]
	chitosan	scaffold	2015	[58]
	chitosan/hydroxyapatite (HAp)	scaffold	2019	[59]
	chitosan	microparticles	2020	[60]
	collagen/chitosan	scaffold	2022	[61]
Cosmetology/ dermatology	chitosan/dextran sulphate	nanoparticles for Pickering emulsion stabilization	2023	[66]
Anti-cancer treatment	chitosan/tripolyphosphate	nanoparticles	2020	[25]
	chitosan/hydroxypropyl methylcellulose cross-linked in the presence of tripolyphosphate	nanoparticles	2021	[75]
	chitosan/tripolyphosphate	nanoparticles	2017	[76]
	chitosan/tripolyphosphate	nanoparticles	2018	[77]
Drug delivery systems	1. lecithin/chitosan; 2. Pluronic^®^ F127/chitosan.	1. nanoparticles; 2. micelles.	2015	[84]
	chitosan	hydrogel	2013	[85]
	chitosan	nanoparticles	2023	[86]
	chitosan	nanoparticles	2021	[87]
	chitosan	nanoparticles	2022	[88]
	chitosan	amphiphilic nanocarrier (ACN)	2021	[89]
Others	Alginate/chitosan	pellets	2020	[92]
	chitosan/carboxymethyl chitosan + carboxymethyl cellulose	film	2020	[94]
	chitosan	solution/film	2024	[95]
	chitosan	solution/film	2023	[96]
	chitosan	solution/film	2023	[97]
	chitosan	solution/film	2022	[98]
	chitosan	solution/film	2023	[99]
	chitosan/sodium tripolyphosphate (TPP)/pectin	particles	2022	[100]
	mesoporous silica nanoparticles (MSN)/chitosan	particles	2022	[101]
	chitosan	film-forming solution	2021	[103]
	alginate/chitosan/β-tricalcium phosphate	hydrogel	2023	[104]
	alginate/chitosan/β-tricalcium phosphate	hydrogel	2020	[105]
